# Case Report: Multiple atherosclerotic plaques at its extreme in synchrony

**DOI:** 10.12688/f1000research.135416.1

**Published:** 2023-06-23

**Authors:** Saket Toshniwal, Isha Sahai, Benumadhab Ghosh, Anuj Chaturvedi, Gajendra Agrawal, Sourya Acharya, Sunil Kumar, Satish Khadse, Kashish Khurana

**Affiliations:** 1 General Medicine, Jawaharlal Nehru Medical College, Datta Meghe Institute of higher education and research, Wardha, Maharashtra, 442001, India; 2 Cardiology, Jawaharlal Nehru medical college, Datta Meghe Institute of Higher Education and Research, Wardha, Maharashtra, 442001, India

**Keywords:** atherosclerosis, subclavian artery stenosis, renal artery stenosis, coronary angiography, dyslipidemia, triple vessel disease, peripheral vascular disease

## Abstract

Peripheral vascular disease in association with renal artery stenosis is an important association which predicts the severity of the disease. An increase in the number of vessels affected by peripheral vascular disease increases the chances of renal artery stenosis. In our case, the patient had primarily presented with anginal chest pain with complaints of claudication which on further investigation was diagnosed to be a triple vessel coronary artery disease along with bilateral subclavian and bilateral renal stenosis. On detailed history taking, risk factors like hypertension and chronic smoking was found to be present in our case which predisposed to peripheral artery disease secondary to atherosclerosis which was diagnosed on further investigations.

Although the association of renal artery stenosis is not very rare in cases of severe peripheral vascular diseases, the presence of a triple vessel coronary artery disease in synchrony is what makes it unique.

Take away lesson from this case report is importance of early diagnosis of dyslipidemia causing atherosclerosis and its complications. Multiple atherosclerotic lesions in synchrony i.e, bilateral renal artery stenosis with bilateral subclavian artery stenosis with coronary artery triple vessel atherosclerotic disease like in our case and its severity should create awareness among health care individuals and early treatment measures including lifestyle modifications should be considered to avoid such drastic events.

## Introduction

Atherosclerosis is a progressive systemic inflammatory disease causing stenotic lesions in the walls of arteries due to the formation of fibrofatty plaque which further predisposes to myocardial infarctions, cerebrovascular events and even disabling peripheral vascular diseases. ^1^ There is an increased prevalence of renal artery stenosis in patients with peripheral vascular disease in 60 years and above age group due to atherosclerosis and also due to the presence of various cardiovascular risk factors. ^2^ Peripheral artery disease (PAD), secondary to atherosclerosis is currently the leading cause of morbidity and mortality in the Western world and its risk factors include age, smoking, hyperlipidemia, and hypertension. ^3^ The outcome of Peripheral vascular disease (PVD) patients is substantially determined by the extent of atherosclerotic comorbidities. ^4^^,^^5^ Renal artery involvement in peripheral artery disease depicts the increased severity of the disease and hence while investigating for peripheral artery disease, renal arteries should be looked for stenotic lesions. ^2^ In a study performed by Aboyans V. *et al,* ^2^ 681 patients who had got their Digital Subtraction Angiography (DSA) done for peripheral artery disease, 14% were found to be prevalent to renal artery stenosis. The association of coronary artery disease in synchrony due to atherosclerosis is rare and not many cases have been reported with multiple atherosclerotic lesions at multiple sites in synchrony like in this case.

In this case, we present a 60-year-old man who presented with anginal-type chest pain and was diagnosed with a triple vessel coronary artery disease who on further evaluation for his claudication of upper limbs was found to have severe peripheral artery disease with bilateral renal artery stenosis.

## Case presentation

A 60-year-old Asian male labourer by occupation, a known hypertensive for 20 years and a chronic smoker for 45 years presented to hospital with anginal-type chest pain radiating to both arms and back with sweating and palpitations associated with breathlessness at rest (NYHA III) with orthopnea and paroxysmal nocturnal dyspnea. The patient also complained of pain in both arms on minimal exertion and while lifting weights which was suggestive of claudication with blackish discolouration of both arms distally.

On general examination, cold pulseless upper extremities with blackish discolouration of distal upper limbs were noted. Axillary, brachial and radial pulses were non-palpable on examination. Other peripheral pulses of temporal, facial, carotid, femoral, popliteal, posterior tibial and dorsalis pedis arteries were palpable and the pulse rate was found to be 80/minute.

On systemic cardiovascular examination, both heart sounds were heard with apex beat felt at 5th intercostal space just lateral to midclavicular line with no murmurs, bilateral air entry was heard with basal crepts bilaterally on respiratory examination. The abdomen was soft and non-tender with no organomegaly, no visible veins, normal peristaltic sounds, no arterial bruit or any abnormal enlargement or a pulsatile mass. The patient was conscious and oriented to time, place and person with no neurological deficits.

An electrocardiograph was done and was suggestive of ST segment depressions in precordial leads with T wave inversions suggesting non ST segment elevation myocardial infarction (NSTEMI).

On routine blood investigations, raised homocysteine levels (75 mcmol/L with normal range of <15 mcmol/L), C - reactive protein (CRP) (6 mg/dL with normal range of <0.3 mg/dL) with raised cardiac biomarkers (CKMB: 50 IU/L and TropI: 24 ng/ml) raised total cholesterol (400 mg/dl with normal range of <170 mg/dl) with Low density lipoprotein (LDL) (140 mg/dl with normal range of <100 ng/dl) with raised triglycerides levels (754 mg/dl with normal range of <200 mg/dl) were observed. cANCA and pANCA with dsDNA and ANA antibodies were also evaluated to rule out vasculitis and were found to be negative. The ankle-brachial index was >1.4 (normal range of 0.9-1.4) which was suggestive of peripheral vascular disease. A CT Aortogram was also done and was not suggestive of aortoarteritis. A diagnosis of peripheral vascular disease with dyslipidemia and raised cardiac biomarkers were made on routine blood investigations and coronary angiography was advised.

**Figure 1.**
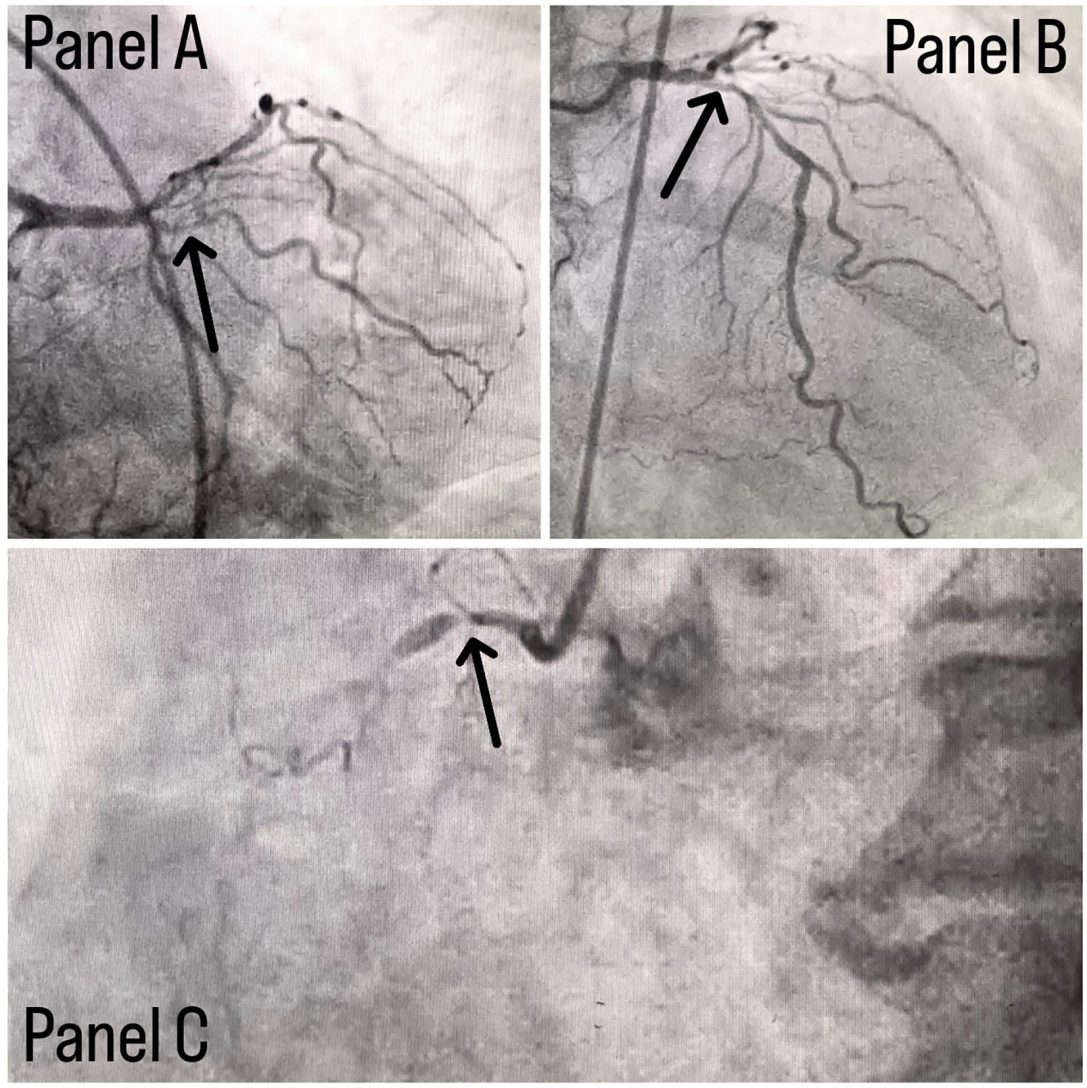
Panel A shows stenosis in proximal LCX with a black arrow, panel B shows stenotic lesion in proximal LAD with a black arrow and panel C shows 100% stenosed RCA with a black arrow.

**Figure 2.**
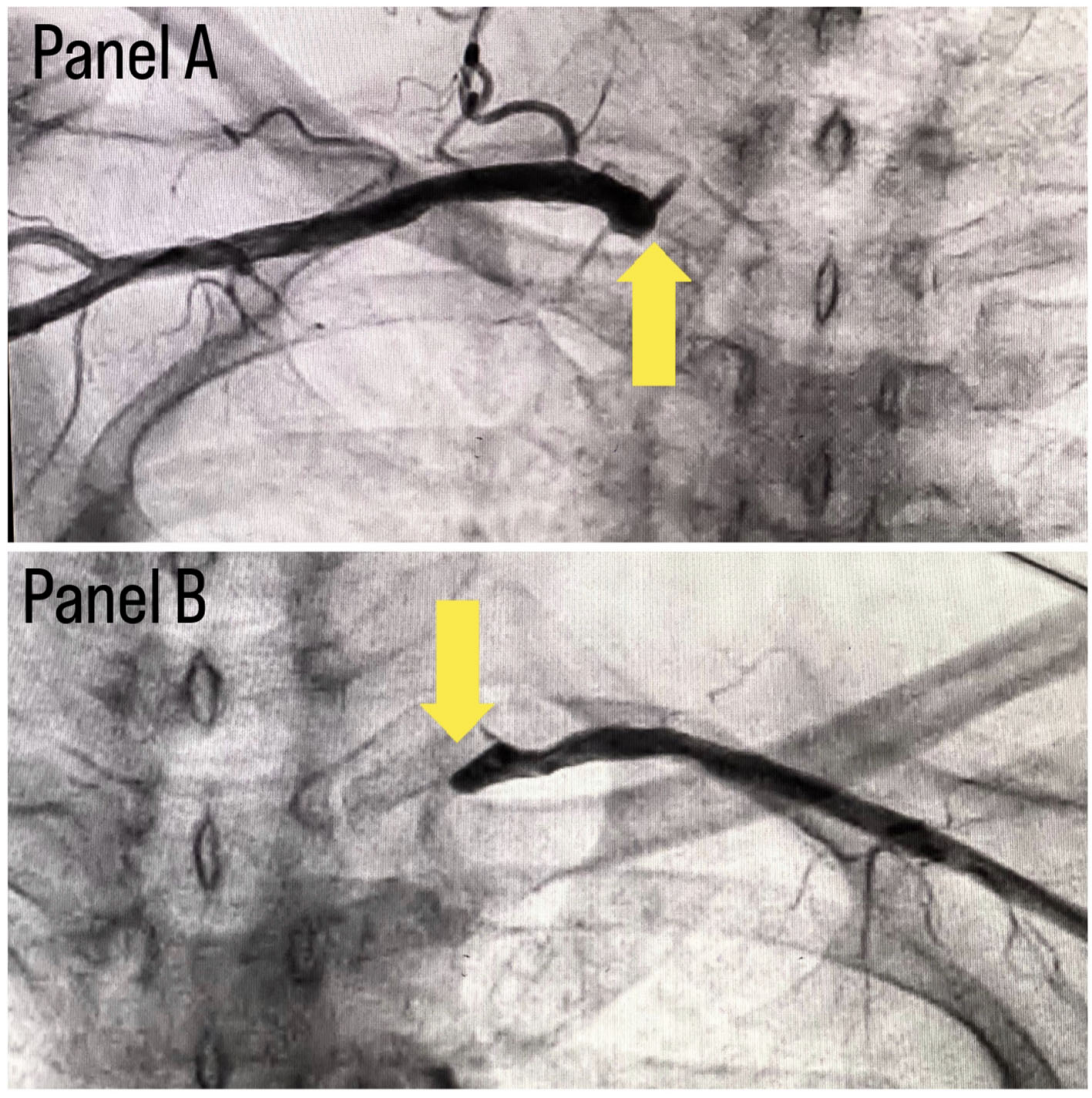
Panel A shows right subclavian stenosis and panel B shows left subclavian stenosis.

**Figure 3.**
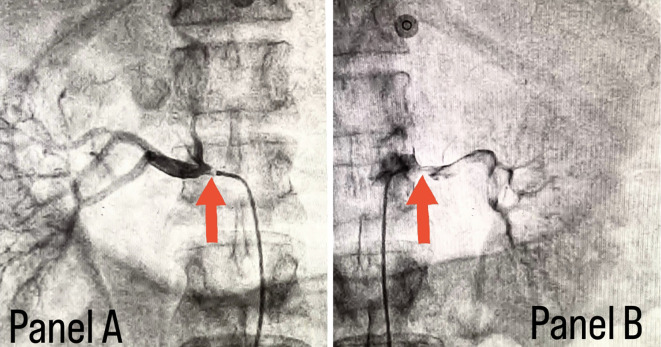
Panel A shows right renal ostial stenosis and panel B shows left renal ostial stenosis marked with red arrows.

Coronary angiography was done and a triple vessel coronary artery disease was diagnosed as shown in Figure 1; in which, panel A shows stenosis in proximal Left circumflex artery (LCX) with a black arrow, panel B shows a stenotic lesion in proximal Left anterior descending artery (LAD) with a black arrow and panel C shows 100% stenosed Right coronary artery (RCA) with a black arrow. A subclavian puncture was taken to look for a subclavian artery considering the claudication symptoms of the patient and peripheral vascular disease was diagnosed with bilateral subclavian artery stenosis as shown in Figure 2, in which panel A shows right subclavian artery stenosis and panel B shows left subclavian artery stenosis marked with yellow arrows. Considering the severity of claudication pain in our case, bilateral renal and femoral arteries were also checked via aortic flush and bilateral renal artery ostial stenosis was seen. Figure 3 panel A shows right renal ostial stenosis and panel B shows left renal ostial stenosis marked with red arrows.

A final diagnosis of atherosclerotic triple vessel coronary artery disease with severe peripheral artery disease with bilateral renal artery stenosis was made. Multiple severe atherosclerotic stenotic lesions in synchrony at multiple sites is what makes it a unique presentation.

The patient was advised coronary artery bypass grafting for his CAD-TVD and was referred to CVTS for further management with dual antiplatelets and statins (Tablet Ecosprin 75 mg one tablet once daily, Tab Clopitab 75 mg one tablet once daily and Tab Rosuvastatin 40 mg one tablet once daily at night to be continued), ACE inhibitors (Tab Ramipril 2.5 mg one tablet twice daily to continue), Beta blockers (Tab Metoprolol 25 mg one tablet twice daily to continue) and other supportive treatment.

## Discussion

Atherosclerosis and coronary artery disease are responsible for a significant number of global fatalities, primarily attributed to heart attacks and congestive heart failure. ^6^ Atherosclerosis evolves due to repetitive damage to the inner lining of blood vessels and the buildup of lipids over time. ^6^ Renal artery stenosis (RAS) frequently occurs as a complication of atherosclerosis and is often connected to chronic heart failure. ^7^ RAS in individuals suffering from CHF may encompass certain clinical features like high blood pressure, deterioration of CHF symptoms, sudden pulmonary oedema, and declining kidney function. ^7^ Renovascular disease often lacks noticeable symptoms, but it can manifest as hypertension, gradual impairment of kidney function, and a significant increase in serum creatinine levels. Other potential clinical presentations may include atheroembolic disease, proteinuria, sudden pulmonary oedema, and chronic heart failure (CHF). ^8^ Peripheral arterial disease, which arises from insufficient blood flow, typically affects the entire cardiovascular system rather than solely the lower extremity blood vessels. Patients displaying symptoms of peripheral arterial disease should undergo an evaluation to assess their risk of atherosclerosis. ^9^ In its milder form, peripheral arterial disease may only cause intermittent claudication, characterized by pain in the lower extremities during physical exertion that subsides at rest. However, when ischemia becomes chronic, critical, or acute, it significantly heightens the risk of gangrene, tissue death, amputation, and premature death. ^9^

In individuals with peripheral vascular disease, renal artery stenosis is frequently observed and is associated with a high mortality rate. Nevertheless, it remains uncertain whether incidentally discovered renal artery stenosis is a risk factor for mortality. ^10^

Different methods, both invasive and non-invasive, can be employed to study atherosclerotic plaques. These methods include intracoronary optical coherence tomography, intravascular ultrasonography, ultrasonography, CCTA, and magnetic resonance imaging. In our study, we discovered that patients with peripheral vascular atherosclerosis (PVA) affecting two to three regions of atherosclerosis, along with coronary stenosis exceeding 50 per cent and high to very high scores of cardiovascular risk, had a significant rate of coronary artery disease (CAD) as found by CCTA.

Cohen et al. conducted a study, ^11^ comparing of the presence of CAD and carotid disease with the help of ultrasonography and CCTA, respectively. They found a strong correlation between carotid plaque and the rigidity of coronary artery calcifications. ^11^ CCTA exhibits high sensitivity in identifying severe stenosis; however, its specificity is inclined to be less, especially in the case of calcified plaques, leading to an overestimation of stenosis. ^12^ CCTA is suitable in patients with a low-to-intermediary risk of CAD to effectively rule out if CAD is present with absolute certainty. However, for patients with high risk for CAD, CCTA is not necessary since it is unable to provide any additional details beyond the already high pre-test probability of CAD. In such cases, subsequent coronary angiography, which remains the gold standard for identifying coronary stenosis, may be required. In recent years, there has been a revived interest in developing new technologies, leading to the expansion of the clinical use of coronary computed tomography angiography (CCTA). ^13^ CCTA, when combined with fractional flow reserve measurement (FFR), offers valuable details for patients having intermediate stenosis. ^13^ This FFR is a measurement that compares the total amount of blood flow in a tapered vessel to the maximum flow threshold in an ordinary vessel.

While non-invasive imaging techniques like brightness-mode USG and magnetic resonance imaging of the heart are utilized to assess early indications of atherosclerosis, they do not offer a comprehensive assessment of the arteries. ^14^ Traditional coronary angiography and imaging for myocardial perfusion also have limitations when it comes to depicting atherosclerosis, particularly in their early phases or if the condition is well-established but still did not yet compromise the stability of the arterial lumen through positive remodelling. We discovered that patients exhibiting plaque vulnerability had significantly elevated levels of LDL-C, total cholesterol, uric acid, and triglycerides in their serum. This finding emphasizes implementing regimen lowering for lipids to mitigate the development of atherosclerotic plaque of the carotid artery. Prophylactic and progressive management is essential to control and manage these conditions.

## Conclusion

Peripheral vascular disease and renal artery stenosis had been found to be associated with one another in more cases than reported. Peripheral vascular disease individually as well as in conjunction with renal artery stenosis present a high risk of death due to cardiovascular disease. Therefore, further emphasis must be given to detailed investigation of all cases of peripheral vascular diseases and renal artery disease for any other associated abnormalities to provide prompt treatment so as to reduce mortality and morbidity in such patients. Early diagnosis of dyslipidemia and the literature depicting the severity of such dyslipidemic conditions causing multiple atherosclerotic lesions in synchrony should create awareness among health care individuals and early treatment measures including lifestyle modifications should be considered to avoid such drastic events. There were very few published studies regarding atherosclerotic triple vessel coronary artery disease with severe peripheral artery disease with bilateral renal artery stenosis in the literature research done for this particular case report. Thus, the identification and publication of such unique reports are equally important to add to the knowledge of medical professionals.

## Consent

Written informed consent for publication of their clinical details and clinical images was obtained from the patient.

## Data Availability

All data underlying the results are available as part of the article and no additional source data are required.
