# Supplementary material for: Case Report: Multiple atherosclerotic plaques at its extreme in synchrony
Source: F1000Res. 2024 Jan 24;12:738. Originally published 2023 Jun 23. [Version 3] doi: 10.12688/f1000research.135416.3 (PMC10905029; doi:10.12688/f1000research.135416.3)
Supplement: Peer review history

# Reviewer report: Reviewer response for version 3

**DOI:** 10.5256/f1000research.161756.r240439

**Published:** 2024-02-15

**Authors:** Daniel Sykora

**Affiliations:** 1 Mayo Clinic School of Graduate Medical Education, Scottsdale, USA

The authors have addressed the outstanding concerns satisfactorily.

Are enough details provided of any physical examination and diagnostic tests, treatment given and outcomes?

Partly

Is the case presented with sufficient detail to be useful for other practitioners?

Partly

Is sufficient discussion included of the importance of the findings and their relevance to future understanding of disease processes, diagnosis or treatment?

No

Is the background of the case’s history and progression described in sufficient detail?

Partly

Reviewer Expertise:

inflammatory cardiomyopathy, cardiac sarcoidosis, peripheral artery disease

I confirm that I have read this submission and believe that I have an appropriate level of expertise to confirm that it is of an acceptable scientific standard.

# Reviewer report: Reviewer response for version 2

**DOI:** 10.5256/f1000research.160282.r233751

**Published:** 2024-01-19

**Authors:** Daniel Sykora

**Affiliations:** 1 Mayo Clinic School of Graduate Medical Education, Scottsdale, USA

Thank you to the authors for revising their manuscript. Major noted changes in the case presentation include greater specificity in the history and physical exam, notably now denoting how acute limb ischemia was ruled out which is a key factor in this patient's management. The authors now sufficiently detail the evidence used to diagnose NSTEMI, as well as present transthoracic echocardiogram data to show the patient has new HFrEF due to ischemic cardiomyopathy. The timeline of events is more clear with sequential description of NSTEMI treatment. The conclusion of the case, in respect to management of the CAD as well as recommendations for management of the other arterial stenoses is now sufficiently clarified. The grammar is now globally improved throughout the text.

 Regarding the discussion, the first paragraph now more appropriately focuses on the systemic manifestations of atherosclerosis.

 There are two more statements in the discussion which warrant revision:

 "In our study, we discovered that patients with peripheral arterial atherosclerosis affecting two to three regions of atherosclerosis, along with coronary stenosis exceeding 50 per cent and high to very high scores of cardiovascular risk, had a significant rate of coronary artery disease (CAD)." -- this sentence cites a 2013 study (Cohen et al.,(2013))[Ref-1]and refers to it as "our study", however there are no common authors between this case report and the cited study in JACC Cardiovascular Imaging. If there are no shared authors, I recommend removing the implication that the cited study was performed by the authors.

 "We discovered that patients exhibiting plaque vulnerability had significantly elevated levels of LDL-C and total cholesterol."  -- it is unclear if this sentence is intended to indicate the authors' reflections on the implications of this current manuscript, or if it is referencing another study by the authors. If the situation is the former, I would recommend removal or rewording of this statement as this case report only reports on a single patient. If the case is the latter, the authors should cite the study they are referring to in this sentence.

Are enough details provided of any physical examination and diagnostic tests, treatment given and outcomes?

Partly

Is the case presented with sufficient detail to be useful for other practitioners?

Partly

Is sufficient discussion included of the importance of the findings and their relevance to future understanding of disease processes, diagnosis or treatment?

No

Is the background of the case’s history and progression described in sufficient detail?

Partly

Reviewer Expertise:

inflammatory cardiomyopathy, cardiac sarcoidosis, peripheral artery disease

I confirm that I have read this submission and believe that I have an appropriate level of expertise to confirm that it is of an acceptable scientific standard, however I have significant reservations, as outlined above.

## References

[1] : Relationship between carotid disease on ultrasound and coronary disease on CT angiography. *JACC Cardiovasc Imaging* .2013;6(11) : 10.1016/j.jcmg.2013.06.007 1160-7 10.1016/j.jcmg.2013.06.007 24229768

# Reviewer report: Reviewer response for version 1

**DOI:** 10.5256/f1000research.148532.r227820

**Published:** 2023-12-15

**Authors:** Daniel Sykora

**Affiliations:** 1 Mayo Clinic School of Graduate Medical Education, Scottsdale, USA

Toshniwal et al. present a case of a 60-year-old male with hypertension and nicotine use evaluated for angina and upper extremity claudication. Initial evaluation is concerning for cardiac and upper extremity ischemia, and objective evaluation with laboratory and electrocardiography raises concern for NSTEMI. Upon undergoing angiography, he is found to have multivessel coronary artery disease, bilateral subclavian and renal artery stenoses. Ankle brachial index studies also revealed PAD with poorly compressible arteries in the lower extremities. Given the severity of his CAD, the patient is referred for bypass grafting and started on medical management with dual antiplatelet therapy, statin, ACE inhibitor, and beta blocker.

 The strengths of this case report include a highly detailed presentation of a patient with multifocal atherosclerosis. The case is interesting and uncommon presentation of multivessel CAD, bilateral upper extremity arterial disease, and bilateral renal artery stenosis. The authors also include high quality angiogram images that add value to the case. This case is an important reminder to all clinicians that atherosclerosis often involves multiple arterial territories.

 The weaknesses of the case report include significant omitted details regarding the history, physical examination, and objective findings. The timeline of the patient’s management is also unclear. The final outcome of the case (outcome of CABG, decisions regarding intervention for his other stenoses) are omitted. Most importantly, much of the discussion appears unrelated to the case report and focuses on CCTA and FFR and implies that this text is a study, instead of a case report.  Due to these concerns, the case presentation and discussion both require crucial substantial revisions, and all the above should be addressed to make this case scientifically sound. The text would also benefit from extensive grammatical and stylistic editing.

 Introduction:

1. The authors use the terms “peripheral artery disease” and “peripheral vascular disease” intermittently in the text. While these terms are often used interchangeably, the authors should use one term only for clarity.
2. The summary statement of the case at the end of the introduction fails to mention the bilateral subclavian artery stenosis, which is an important finding in the setting of upper extremity claudication.

Case presentation:

1. The past medical history of the patient is described but baseline medications are not mentioned. It would be valuable to know if the patient was taking antihypertensive medications at baseline and the degree of control of hypertension, as treatment refractory hypertension is a common manifestation of renal artery stenosis, which is later diagnosed in this patient.
2. The acuity of the chest pain is not mentioned. Given the clinical concern later mentioned in the case report for NSTEMI, the acuity and time course of the chest pain should be described.
3. Likewise, the time course of the upper extremity claudication symptoms should be described, as should the presence/absence of lower extremity claudication symptoms (especially given later confirmed lower extremity PAD with poorly compressible arteries).
4. The patient is described as having NYHA Class III heart failure symptoms, but the text does not mention if assessment of his cardiac function was later performed. If investigated, the authors should describe if the patient had heart failure and how this was diagnosed, especially since the discussion of this paper extensively mentions CHF.
5. The authors should mention whether the patient had cerebrovascular symptoms given the possibility of vascular steal syndromes in the setting of later discovered subclavian artery stenosis.
6. The “general” and “systemic cardiovascular examination” sections should be combined into a single physical examination section, especially since the current “general examination” section details pulse examination.
7. The authors describe “cold pulseless upper extremities with blackish discolouration of distal upper limbs” and note absence of “axillary, brachial and radial pulses.” These initial physical exam findings are concerning for acute limb ischemia. The authors should describe how acute limb ischemia was ruled out (e.g. were pulses subsequently detected via Doppler assessment?).
8. The authors should use the term appropriate medical terminology “cyanosis” as opposed to “blackish discolouration” when describing their physical exam findings.
9. The word “electrocardiograph” should instead read “electrocardiogram.”
10. The description of the ECG should be more detailed, at minimum including description of which leads showed ST segment depressions and T wave inversions. If space permits, the ECG should be added as a figure.
11. The authors mention the ECG results are concerning for NSTEMI. As NSTEMI diagnosis necessitates the presence of dynamic cardiac biomarkers, this should be rephrased to “suggesting acute myocardial ischemia.”
12. The paragraph which begins with “On routine blood investigations…” requires significant revision. In the prior paragraph, the authors mention their suspicion for NSTEMI based on ECG results. Therefore, this paragraph should focus on the initial laboratory investigations pertinent to the concern for NSTEMI that would have informed immediate management decisions, namely cardiac biomarkers. The authors mention the CK-MB and troponin I, but do not mention whether these biomarkers were trended over time and whether dynamic changes were observed. This is crucial to know to confirm the diagnosis of NSTEMI. The patient’s creatinine and estimated GFR should be mentioned in this paragraph, given the later diagnosed bilateral renal artery stenosis.
13. CK-MB and troponin I reference ranges should be mentioned.
14. The timeline of the other studies mentioned (homocysteine, CRP, cholesterol, vasculitis labs, ABI, CT aortogram) in relation to the coronary angiogram is unclear. If the authors were concerned for NSTEMI, the timeline of angiography after admission should be made clear. If other studies were pursued later in the care of this patient, they should be mentioned later in the text. If the concern was for NSTEMI, the priority of these studies seems lower than coronary angiogram so it is confusing to present them earlier in the text.
15. If the patient was diagnosed with NSTEMI, the management prior to angiography should be described. Did the patient receive loading doses of aspirin, clopidogrel, or start on a heparin infusion? These details should be mentioned.
16. The ABI should be described for both extremities.
17. The authors are correct in stating that ABI > 1.4 is suggestive of peripheral artery disease, but this finding is usually seen in poorly compressible arteries due to chronic kidney disease or diabetes mellitus, as opposed to atherosclerotic lower extremity PAD. Whether the patient had these comorbidities should be mentioned.
18. The repetition of the figure descriptions for Figure 1,2, and 3, (e.g. description of arrows) in the text is redundant and can be removed.
19. The results of the femoral artery angiography are not mentioned in the text and should be added.
20. The degree of multifocal atherosclerosis seen in this patient at his age is impressive, and it would be helpful to mention whether he had any family history of early coronary artery disease or whether any laboratory testing for inherited atherosclerosis risk factors (e.g. Lipoprotein(a)).
21. The sentence “Multiple severe atherosclerotic stenotic lesions in synchrony at multiple sites is  what makes it a unique presentation” should be removed from the case presentation as this section should focus on presentation of objective data regarding this case as opposed to author interpretation, which should be reserved for the discussion.
22. The abbreviations “CAD-TVD” and “CVTS” are not defined elsewhere in the text and should be either defined or removed (preferably, as there are not universally recognized abbreviations).
23. Generic medication names should be used (aspirin and clopidogrel) instead of trade names for ease of readability.
24. The specific type of metoprolol used should be specified (e.g. Metoprolol tartrate).
25. Based on the history provided, the patient had evidence of (at least) severely symptomatic chronic bilateral upper extremity limb ischemia. Was endovascular intervention for the subclavian artery stenoses considered? If not, was surgical revascularization later considered or pursued?
26. Likewise, the rationale behind pursuing medical management only (as opposed to stenting) for the renal artery stenosis should be mentioned.
27. The outcome of the case, including the outcome of CABG and need for any subsequent procedures, should be added.
28. The blood pressure and cholesterol target goals for this patient should be mentioned since the patient was started on statin and antihypertensive therapy.

Discussion:

1. The term “heart attacks” in the first paragraph should be replaced with more appropriate medical terminology: “myocardial infarction.”
2. The first paragraph focuses on the relationship between RAS and heart failure, but this patient was never diagnosed with heart failure. This should be either removed, or the patient’s diagnosis of heart failure should be clarified in the case presentation section.
3. This discussion does not fit the content of the case presentation. For example, consider the sentence: “In our study, we discovered that patients with peripheral vascular atherosclerosis (PVA) affecting two to three regions of atherosclerosis, along with coronary stenosis exceeding 50 per cent and high to very high scores of cardiovascular risk, had a significant rate of coronary artery disease (CAD) as found by CCTA.” This sentence appears unrelated to the case report as CCTA is not mentioned in the case presentation. A large portion of the discussion also focuses on CCTA and FFR, which is not relevant to the case and was not used in the diagnosis of this patient, who proceeded directly to coronary angiogram.
4. In the last paragraph of the discussion, the sentence “We discovered that patients exhibiting plaque vulnerability had significantly elevated levels of LDL-C, total cholesterol, uric acid, and triglycerides in their serum” is also unrelated to the case presentation.

Are enough details provided of any physical examination and diagnostic tests, treatment given and outcomes?

Partly

Is the case presented with sufficient detail to be useful for other practitioners?

Partly

Is sufficient discussion included of the importance of the findings and their relevance to future understanding of disease processes, diagnosis or treatment?

No

Is the background of the case’s history and progression described in sufficient detail?

Partly

Reviewer Expertise:

inflammatory cardiomyopathy, cardiac sarcoidosis, peripheral artery disease

I confirm that I have read this submission and believe that I have an appropriate level of expertise to state that I do not consider it to be of an acceptable scientific standard, for reasons outlined above.

# Response

**Published:** 2024-01-01

**Authors:** Saket Toshniwal

**Affiliations:** General medicine, datta meghe institute of higher education and research, wardha, Wardha, Maharashtra, India

Respected sir,

 Thank you for reviewing my article and adding your valuable inputs.

 The article has been revised according to the instructions and they are as follows

**Revisions:**

**Introduction**

1. The interchangeable terms 'peripheral artery disease' and 'peripheral vascular disease' have been rectified and only one term has been used i.e; 'peripheral artery disease'
2. 'Subclavian artery stenosis' has been added to the introduction section.

**Case presentation**

1. Past history of the patient has been revised and more detailed past history has been added to the text with comment on degree of control of hypertension ​​​​​​.
2. Acuity of chest pain has been added along with description of chest pain and time course
3. Time course of the upper extremity claudication symptoms has been added with comment on lower limb claudication symptoms.
4. Heart failure was diagnosed on 2d echocardiogram and this has been added to the text.
5. The patient had no neurological deficits or any cerebrovascular symptoms which has been added to the text.
6. General and systemic examination sections have been combined.
7. Acute limb ischemia was diagnosed via doppler assessment after clinically suspecting it. This has been added to the text.
8. 'Blackish discolouration' has been replaced with 'cyanosed' while describing physical examination.
9. 'Electrocardiograph' has been replaced with 'electrocardiogram'
10. ECG has been described in details as instructed
11. NSTEMI rephrased as "acute myocardial ischemia' as instructed
12. The paragraph starting with "On routine blood investigations..." has been revised and structured as instructed. Cardiac biomarkers has been mentioned before routine investigations which lead to the diagnosis of NSTEMI. Also, serum creatinine and estimated GFR have been mentioned in subsequent paragraphs.
13. Reference range for CKMB and TROP-I have been mentioned
14. The timeline of the study in the article has been revised as instructed and cardiac biomarkers and cardiac evaluation following coronary angiography have been mentioned at first.
15. Management prior to angiography has been added.
16. ABI for both extremities have been mentioned.
17. ABI has been revised and mentioned in the text.
18. The repetition of figure description in the text has been removed.
19. Results of femoral artery angiography has been added.
20. Inherited atherosclerotic factors (Lipoprotein a ) has been ruled out, with non significant family history's been added to the text.
21. The sentence " multiple severe atherosclerotic stenotic lesions in synchrony at multiple sites is what makes it a unique presentation" has been removed from case presentation section.
22. Abbrevations such as CAD-TVD and CVTS has been removed.
23. Generic medication names has been used.
24. Specific type of metoprolol detail has been added
25. "Endovascular intervention was considered and advised to the patient but were not willing for the same" added to the text.
26. Renal artery endovascular stunting was advised but the patient was not willing for the same.
27. Outcome of the case couldn't be tracked as the patient failed to maintain follow up. This has been added to the text.
28. Target blood pressure and cholesterol levels have been mentioned.

**Discussion:**

1. "Heart attacks" have been replaced with "myocardial infarction"
2. The patient was diagnosed to have heart failure with reduced ejection fraction which has been added in the case presentation section.
3. The sentence "In our study, we discovered that patients with peripheral vascular atherosclerosis (PVA) affecting two to three regions of atherosclerosis, along with coronary stenosis exceeding 50 per cent and high to very high scores of cardiovascular risk, had a significant rate of coronary artery disease (CAD) as found by CCTA" has been removed. CCTA and FFR though not used in the diagnosis of our case has been given importance in the discussion section in view of advancement in diagnosis with newer modalities and to shed light upon the literature related to it for the practitioners.
4. The sentence in the last paragraph has been revised.

Kindly review the revisions made.

 Thank you

 Yours sincerely,

 Dr. Saket Toshniwal

# Reviewer report: Reviewer response for version 1

**DOI:** 10.5256/f1000research.148532.r181682

**Published:** 2023-11-10

**Authors:** Vishakha Jain

**Affiliations:** 1 Department of Medicine, All India Institute of Medical Sciences (AIIMS), Bibinagar, Hyderabad, Telangana, India

I must congratulate the authors on a well written case report with relevant information about the existing literature and good discussion points.

 The topic under consideration is very important and highlights the need of screening patients with PAD for CAD/coronary stenosis as well, the ABI has been a good tool for the same.

Are enough details provided of any physical examination and diagnostic tests, treatment given and outcomes?

Yes

Is the case presented with sufficient detail to be useful for other practitioners?

Yes

Is sufficient discussion included of the importance of the findings and their relevance to future understanding of disease processes, diagnosis or treatment?

Yes

Is the background of the case’s history and progression described in sufficient detail?

Yes

Reviewer Expertise:

Non communicable diseases , Diabetes mellitus , CAD , NAFLD , medical education , Echocardiography

I confirm that I have read this submission and believe that I have an appropriate level of expertise to confirm that it is of an acceptable scientific standard.
